# Supplementary material for: DNMT3B PWWP mutations cause hypermethylation of heterochromatin
Source: EMBO Rep. Author manuscript; Available in PMC 2024 Mar 12. (PMC7615734; doi:10.1038/s44319-024-00061-5)
Supplement: Supplementary material [file EMS194008-supplement-Supplementary_material.pdf]

Taglini *et al*., Appendix of supplementary materials

Contents

Appendix table S1	page 2
Appendix table S2	page 2
Appendix table S3	page 3
Appendix table S4	page 4
Appendix table S5	page 5
Appendix table S6	page 7
Appendix figure S1	page 8
Appendix figure S2	page 11
Appendix figure S3	page 14
Appendix figure S4	page 16
Appendix figure S5	page 18
Appendix figure S6	page 20
Appendix figure S7	page 22
Appendix figure S8	page 24

Appendix Table S1

Oligonucleotides used in this study.

CRISPR/Cas9-edited HCT116 cell lines	
**Name**	**Sequence**
DNMT3B-EX7-W263A-HDR-Template	CAAGCGACAGGCTATGTCTGGCATGCGATGGGTCCAGGCGTTTGGCGATGGCAAGTTCTCCGAGGTGAGTCCGGG GAAGG
gRNA-EX7-T	[Phos]CACCGACTTGCCATCGCCAAACCAC
gRNA-EX7-B	[Phos]AAACGTGGTTTGGCGATGGCAAGTC
DNMT 3B-EX7-screen-F	TTTGGAATAGGGGACCTCGTGTGG
DNMT 3B-EX7-screen-R	CACACATCTGCAGAATACAATCCCAGG
**bsPCR primers**	
**Name**	**Sequence**
bs-BRCA2-UMI-3-F	TCGTCGGCAGCGTCAGATGTGTATAAGAGACAGNNNNGTTTGGGATTTTTAAGGGTTAG
bs-BRCA2-UMI-3-R	GTCTCGTGGGCTCGGAGATGTGTATAAGAGACNNNNCTCCAAAAATACAATTCTTTTT
bsH3K9me3L1-UMI-F	TCGTCGGCAGCGTCAGATGTGTATAAGAGACAGNNNNGAGTTTAGTATTTTTTTTCGGAGTT
bsH3K9me3L1-UMI-R	GTCTCGTGGGCTCGGAGATGTGTATAAGAGACNNNNCCCTCCTCCTTAAAATAAAAAAAAC
bsH3K9me3L2-UMI-F	TCGTCGGCAGCGTCAGATGTGTATAAGAGACAGNNNNGAAGAGGAGGGTGGTTGGGAAGGGTG
bsH3K9me3L2-UMI-R	GTCTCGTGGGCTCGGAGATGTGTATAAGAGACNNNNCATAATCACATAAACAAAAACCACAA

Appendix Table S2

Summary of sequencing statistics for WGBS. Aligned reads counts are following PCR duplicate removal.

Sample	Total reads (x10^6)	Aligned reads (x10^6)	Mean CG coverage	Bisulfite conversion rate
3BKO vs HCT116 and expression of 3B and 3Bcd in 3BKO cells - Figure 1 and S1				
HCT116	43.40	32.72	2.53	99.28
3BKO	52.38	39.73	2.75	99.28
3BKO+3B	61.34	47.06	2.66	99.28
3BKO+3Bcd	48.45	36.89	2.53	99.28
expression of 3A and 3BW263A in 3BKO cells - Figure 2 and S2				
HCT116	84.54	64.46	2.55	99.70
3BKO	116.48	90.62	3.11	99.69
3BKO+3B	94.42	72.72	2.67	99.68
3BKO+3BW263A	91.77	69.95	2.57	99.69
3BKO+3A	116.63	90.44	3.02	99.67
3BKO+3Acd	92.15	70.65	2.54	99.57
control and 3BW263A CRISPR clones - Figure 2 and S3				
HCT116	44.07	43.03	2.02	99.64
W263A c1	58.61	57.72	2.43	99.68
W263A c2	60.38	59.64	2.50	99.70
W263A c3	70.46	69.78	2.74	99.66
control c1	87.63	86.98	3.18	99.67
control c2	69.60	68.79	2.68	99.66
expression of 3BΔN in 3BKO cells- Figure S5 and S7				
HCT116	45.08	35.72	1.88	99.68
3BKO	49.79	39.44	2.01	99.64
3BKO+3B rep.1	46.77	36.77	1.97	99.52
3BKO+3BΔN rep.1	47.28	37.05	1.99	99.58
3BKO+3B rep.2	50.25	39.81	2.01	99.60
3BKO+3BΔN rep.2	46.77	36.90	1.98	99.57

Appendix Table S3

Summary of sequencing statistics for ChIP-seq and ChIP-Rx-seq. Aligned reads are after duplicate and multi-mapper removal and for H3K36me3 and T7-DNMT3B ChIP-Rx-seq reported for both genomes (hg38/dm6). For T7-DNMT3B ChIP-Rx-seq, which was paired end sequencing, fragments are quoted.

Sample	Total reads or read pairs (x10^6)	Aligned reads or fragments hg38 (x10^6) or hg38 (x10^6)/dm6 (x10^3)
	H3K9me3 and H3K27me3	
HCT116 Input rep. 1	41.49	32.98
DNMT3B KO Input rep. 1	55.10	43.87
HCT116 H3K9me3 rep. 1	37.95	29.60
DNMT3B KO H3K9me3 rep. 1	34.10	26.71
HCT116 H3K27me3 rep. 1	52.57	40.43
DNMT3B KO H3K27me3 rep. 1	35.81	28.57
HCT116 Input rep. 2	50.59	39.79
DNMT3B KO Input rep. 2	53.15	42.16
HCT116 H3K9me3 rep. 2	29.01	22.61
DNMT3B KO H3K9me3 rep. 2	45.66	35.86
HCT116 H3K27me3 rep. 2	51.08	40.08
DNMT3B KO H3K27me3 rep. 2	60.89	48.65
	H3K36me3	
HCT116 Input rep. 1	70.54	59.19/110
HCT116 H3K36me3 rep. 1	70.07	58.09/290
DNMT3B KO Input rep. 1	63.62	53.71/100
DNMT3B KO H3K36me3 rep. 1	66.01	54.92/250
HCT116 Input rep. 2	72.15	60.51/130
HCT116 H3K36me3 rep. 2	70.96	58.78/280
DNMT3B KO Input rep. 2	67.03	56.62/110
DNMT3B KO H3K36me3 rep. 2	67.84	56.57/220
	H3K4me3	
HCT116 Input rep. 1	107.75	84.71
DNMT3B KO Input rep. 1	74.93	59.03
HCT116 H3K4me3 rep. 1	83.08	64.17
DNMT3B KO H3K4me3 rep. 1	51.52	40.69
HCT116 Input rep. 2	58.10	45.77
DNMT3B KO Input rep. 2	33.20	25.93
HCT116 H3K4me3 rep. 2	95.11	72.07
DNMT3B KO H3K4me3 rep. 2	79.37	60.17
	endogenous T7-DNMT3B	
HCT116 (mock) Input rep. 1	74.80	63.42/5
HCT116 (mock) T7-IP rep. 1	52.35	39.36/1902
T7-DNMT3B Input rep. 1	78.31	66.45/5
T7-DNMT3B T7-IP rep. 1	73.32	62.90/332
HCT116 (mock) Input rep. 2	74.94	63.58/6
HCT116 T7-IP (mock) rep. 2	66.10	56.25/85
T7-DNMT3B Input rep. 2	68.89	57.81/6
T7-DNMT3B T7-IP rep. 2	83.90	70.50/179
	T7-DNMT3B in WT cells	
HCT116 (mock) Input rep. 1	34.21	25.62
HCT116 +T7-DNMT3B Input rep. 1	50.94	37.63
HCT116 (mock) T7-IP rep. 1	32.82	24.67
HCT116 +T7-DNMT3B T7-IP rep. 1	52.42	39.48
HCT116 (mock) Input rep. 2	66.06	47.61
HCT116 +T7-DNMT3B Input rep. 2	39.87	29.26
HCT116 (mock) T7-IP rep. 2	28.86	21.65
HCT116 +T7-DNMT3B T7-IP rep. 2	37.13	28.05
	T7-DNMT3B in 3BKO cells	
3BKO (mock) Input rep. 1	69.38	49.07/3.15
3BKO +T7-DNMT3B Input rep. 1	89.62	61.21/4.93
3BKO +T7-DNMT3BW263A Input rep. 1	108.42	73.43/4.36
3BKO +T7-DNMT3BΔN Input rep. 1	92.92	64.51/2.23
3BKO (mock) T7-IP rep. 1	53.30	38.06/33.96
3BKO +T7-DNMT3B T7-IP rep. 1	81.88	57.86/10.79
3BKO +T7-DNMT3BW263A T7-IP rep. 1	76.48	53.87/14.16

3BKO +T7-DNMT3BΔN T7-IP rep. 1	81.00	58.17/6.33
3BKO (mock) Input rep. 2	71.84	49.91/3.28
3BKO +T7-DNMT3B Input rep. 2	94.73	65.36/2.32
3BKO +T7-DNMT3BW263A Input rep. 2	89.59	62.45/1.60
3BKO +T7-DNMT3BΔN Input rep. 2	87.19	61.60/1.52
3BKO (mock) T7-IP rep. 2	65.52	45.72/34.06
3BKO +T7-DNMT3B T7-IP rep. 2	77.87	56.56/8.73
3BKO +T7-DNMT3BW263A T7-IP rep. 2	73.14	52.74/9.73
3BKO +T7-DNMT3BΔN T7-IP rep. 2	73.46	53.63/5.67

Appendix Table S4

Summary of sequencing statistics for RNA-seq. Aligned fragments are after duplicate and multi- mapper removal.

Sample	Total fragments (x10^6)	Aligned fragments (x10^6)
DNMT3B KO rep. 1	177.22	146.38
DNMT3B KO rep. 2	147.86	121.53
DNMT3B KO rep. 3	166.99	138.99
HCT116 rep. 1	181.23	148.20
HCT116 rep. 2	173.24	140.81
HCT116 rep. 3	144.21	119.60

Appendix Table S5

Summary of bisulfite PCR reads following PCR duplicate removal.

Sample	Locus	Number of reads
	D266A experiment	
3BKO	BRCA2	15
3BKO+3B		10
3BKO+3BW263A		7
3BKO+3BD266A		16
3BKO+eGFP		11
3BKO	H3K9me3 locus 1	56
3BKO+3B		43
3BKO+3BW263A		44
3BKO+3BD266A		56
3BKO+eGFP		24
3BKO	H3K9me3 locus 2	156
3BKO+3B		148
3BKO+3BW263A		166
3BKO+3BD266A		196
3BKO+eGFP		162
	S270P experiment	
3BKO	BRCA2	22
3BKO+3B		22
3BKO+3BW263A		7
3BKO+3BS270P		13
3BKO+eGFP		12
3BKO	H3K9me3 locus 1	84
3BKO+3B		75
3BKO+3BW263A		22
3BKO+3BS270P		105
3BKO+eGFP		43
3BKO	H3K9me3 locus 2	140
3BKO+3B		145
3BKO+3BW263A		155
3BKO+3BS270P		141
3BKO+eGFP		202
	PWWP and ΔN mutants experiment	
3BKO	BRCA2	101
3BKO+3B		55
3BKO+3BW263A		38
3BKO+3BK276E		77
3BKO+3BK294E		64
3BKO+3BΔPWWP		75
3BKO+3BΔN		66
3BKO+3BΔN W263A		100
3BKO+eGFP		46
3BKO	H3K9me3 locus 1	1652
3BKO+3B		797
3BKO+3BW263A		1078
3BKO+3BK276E		1449
3BKO+3BK294E		695
3BKO+3BΔPWWP		605
3BKO+3BΔN		1198
3BKO+3BΔN W263A		1025
3BKO+eGFP		791
3BKO	H3K9me3 locus 2	5363
3BKO+3B		4772
3BKO+3BW263A		4332
3BKO+3BK276E		5161
3BKO+3BK294E		4157
3BKO+3BΔPWWP		4979
3BKO+3BΔN		5191
3BKO+3BΔN W263A		5135
3BKO+eGFP		4035
	EF1α - lower expressing promoter experiment	
3BKO	BRCA2	795

3BKO+3B		893
3BKO+3Bcd		1020
3BKO+3BW263A		914
3BKO+eGFP		767
3BKO	H3K9me3 locus 1	2313
3BKO+3B		3203
3BKO+3Bcd		3093
3BKO+3BW263A		3244
3BKO+eGFP		3014
3BKO	H3K9me3 locus 2	4015
3BKO+3B		3994
3BKO+3Bcd		4160
3BKO+3BW263A		4333
3BKO+eGFP		3566
W263Acd experiment		
3BKO	BRCA2	497
3BKO+3B		445
3BKO+3Bcd		281
3BKO+3BW263A		168
3BKO+3BW263Acd		225
3BKO+eGFP		634
3BKO	H3K9me3 locus 1	1974
3BKO+3B		1701
3BKO+3Bcd		1257
3BKO+3BW263A		718
3BKO+3BW263Acd		630
3BKO+eGFP		2708
3BKO	H3K9me3 locus 2	5723
3BKO+3B		5423
3BKO+3Bcd		4712
3BKO+3BW263A		5279
3BKO+3BW263Acd		5513
3BKO+eGFP		7228

Appendix Table S6

Recombinant DNMT3B expression constructs

Construct	Protein (aa)	Domain(s)	Mutations
His-MBP-DNMT3B PWWP	213-351	PWWP	-
His-MBP-DNMT3B PWWP K251E R252E K276E K294E	213-351	PWWP	K251E R252E K276E K294E
His-MBP-DNMT3B PWWP K294E	213-351	PWWP	K294E
His-MBP-DNMT3B PWWP K276E	213-351	PWWP	K276E
His-MBP-DNMT3B PWWP-ADD	213-555	PWWP-ADD	-
His-MBP-DNMT3B PWWP-ADD S270P	213-555	PWWP-ADD	S270P
His-MBP-DNMT3B PWWP-ADD W263A	213-555	PWWP-ADD	W263A
His-MBP-DNMT3B N-PWWP	1-351	N-PWWP	-
His-MBP-DNMT3B N-PWWP-ADD	1-555	N -PWWP-ADD	-
His-MBP-DNMT3B N	1-205	N terminal region	-
His-MBP-DNMT3B ADD	412-555	ADD	-

Appendix Figure S1

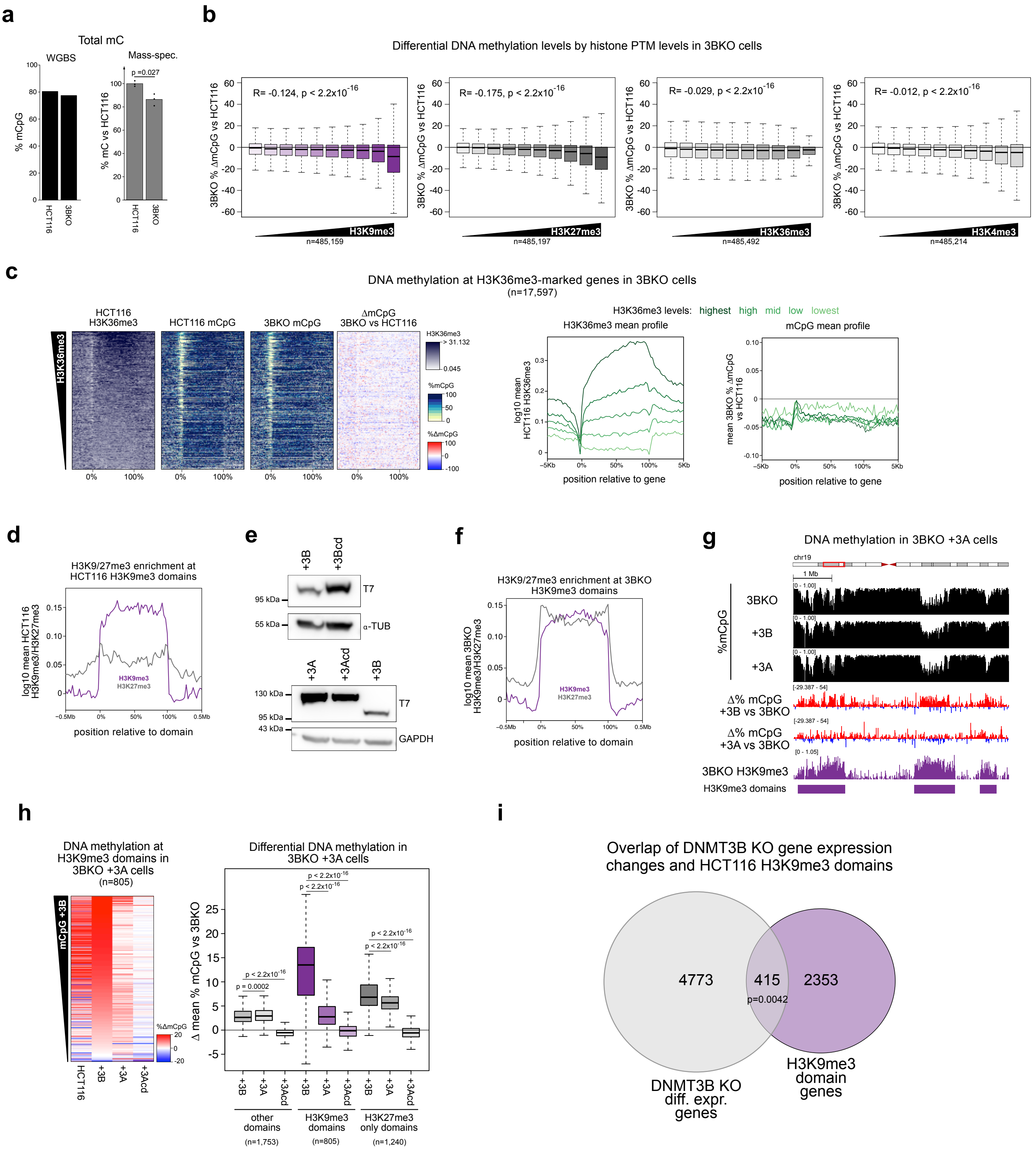

Appendix Figure S1

DNMT3B methylates heterochromatin

a) Total DNA methylation levels are reduced in DNMT3B KO cells. Barplot of total methylated cytosine levels estimated by WGBS (left) and mass-spectrometry (right, mean methylation levels relative to HCT116 cells from 3 technical replicates). P- values are from two-sided T-tests. **b)** Correlation between loss of DNA methylation and enrichment of chromatin marks in DNMT3B KO cells. Boxplots showing difference in DNA methylation in DNMT3B KO to HCT116 cells at 2.5 kb genomic windows divided in deciles according to their histone modification enrichment calculated in HCT116 cells. The Pearson's correlation coefficient (R) is shown alongside its associated p-value. In each case n is the number of windows analysed and is shown below the plots. **c)** H3K36me3 enrichment at gene bodies does not correlate with DNA methylation loss in DNMT3B KO cells. Left, heatmaps showing levels of H3K36me3 and of absolute and differential DNA methylation at gene bodies in HCT116 and DNMT3B KO cells. Genes are ranked by their mean H3K36me3 levels (n=17,597 genes). Right, profiles of H3K36me3 and differential DNA methylation levels at gene bodies, divided in 5 equally sized groups according to their H3K36me3 enrichment in HCT116 cells. **d)** Mean H3K9me3 and H3K27me3 ChIP-seq profiles at H3K9me3 domains in HCT116 cells (mean from n=545 domains). 82.9% of HCT116 H3K9me3 domains overlap with one or more H3K27me3 domains. **e)** Western blots showing ectopic expression of T7-tagged DNMT3B, DNMT3Bcd, DNMT3A and DNMT3Acd in DNMT3B KO cells. **f)** Mean H3K9me3 and H3K27me3 ChIP-seq profiles at H3K9me3 domains in DNMT3B KO cells (mean from n=796 domains). 92.6% of DNMT3B KO H3K9me3 domains overlap with one or more H3K27me3 domain. **g, h)** DNMT3B remethylates heterochromatin to a significantly higher level than DNMT3A. **g)** Representative genomic location showing gains of DNA methylation at H3K9me3 domains in DNMT3BKO cells expressing DNMT3B or DNMT3A. Genome browser plots show absolute (black) and differential (gain=red, loss=blue) DNA methylation levels, DNMT3B KO ChIP-seq signals and H3K9me3 domains defined in DNMT3B KO cells. ChIP-seq are normalised reads per 10^6. **h)** Left, heatmaps of relative methylation levels at H3K9me3 domains (n=805 domains). Values denote the change in methylation relative to DNMT3B KO cells. H3K9me3 domains are defined in DNMT3B KO cells and ranked by the mean gain of DNA methylation in DNMT3B KO cells expressing DNMT3B. Left, boxplots of DNA methylation difference to DNMT3B KO cells at H3K9me3 (n=805 domains), H3K27me3-only marked domains (n=1,240) and the rest of the genome (other, n=1,735). +3B = DNMT3B KO + DNMT3B; +3A = DNMT3B KO + DNMT3A; +3Acd = DNMT3B KO + catalytically dead DNMT3A. **i)** Venn diagram of the overlap between differentially expressed genes in DNMT3B KO cells and genes located in HCT116 H3K9me3 domains. P-value is from a Fisher's exact test. For boxplots in **b.** and **h.**: lines = median; box = 25th–75th percentile; whiskers = 1.5 × interquartile range from box. P-values in **h.** are from two-

sided Wilcoxon rank sum tests. All histone and T7-DNMT3B ChIP-seq data shown are derived from the mean of 2 biological replicates.

Appendix Figure S2

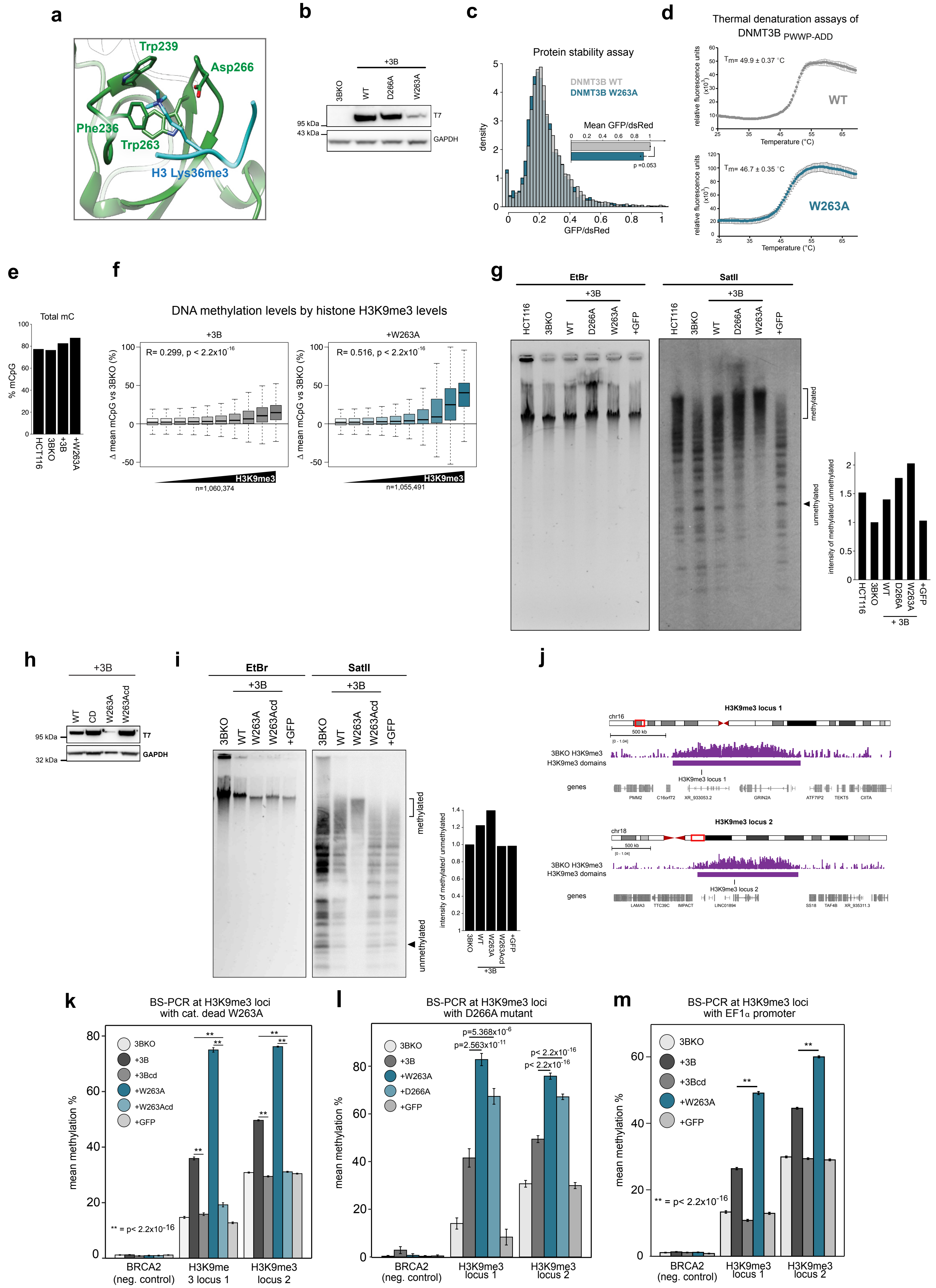

Appendix Figure S2

Mutation of DNMT3B H3K36me3 binding residues causes gains of DNA methylation at heterochromatin

a) Magnified view of DNMT3B ribbon representation of aromatic cage structure bound to trimethyl lysine 36 of H3 (blue) (Protein Data Bank code 5CIU). **b-d)** DNMT3B^{W263A} is stable. **b)** Western blot of ectopically expressed T7-tagged DNMT3B proteins in DNMT3B KO cells. **c)** Stability of DNMT3B^{WT} and DNMT3B^{W263A} measured by fluorescence reporter. Histogram shows the density distribution of single cell GFP/dsRed ratios of one representative experiment. The barplot shows the mean GFP/dsRed ratios of all the cells, measurements for the mutant normalized to the wild-type, for three independent experiments. P value is from student's T-test. **d)** Thermal denaturation assays on purified DNMT3B^{WT} or DNMT3B^{W263A} protein PWWP and ADD fragments. Graphs showing SYBR-safe fluorescence measured at temperatures from 25°C to 69.5°C. Mean and standard deviation of three technical replicates are plotted with mean Tm values stated. DNMT3B^{WT} data are repeated from *Fig 4e* as the data were part of the same experiment. **e)** Total levels of DNA methylation measured by WGBS analysis. +3B = DNMT3B^{WT} cells; +W263A = DNMT3B^{W263A} cells. **f)** Boxplots showing gains of DNA methylation in DNMT3B^{WT} or DNMT3B^{W263A} cells at 2.5 kb genomic windows (n=1,060,374 and 1,055,491 windows respectively) ranked according to H3K9me3 enrichment in DNMT3B KO cells (derived from the mean of 2 biological replicates) before being grouped into deciles of an equal size based on rank. Lines = median; box = 25th–75th percentile; whiskers = 1.5 × interquartile range from box. Pearson's correlations, R, and associated p-values are shown. **g)** Methylation sensitive Southern blot showing reduced digestions of satellite II sequences in DNMT3B^{W263A} or DNMT3B^{D266A} cells compared to DNMT3B^{WT}, DNMT3B KO cells and DNMT3B KO cells expressing GFP. EtBr is shown as loading control. Barplot shows signal quantification of satellite II Southern blot using the ratio of the methylated over unmethylated regions indicated. **h-k)** DNMT3B^{W263Acd} fails to methylate heterochromatin. **h)** Western blot of ectopically expressed T7-tagged DNMT3B proteins in DNMT3B KO cells. **i)** Methylation sensitive Southern blot showing similar digestions of satellite II sequences in DNMT3B^{W263Acd} and DNMT3B KO cells expressing GFP, in contrast with protection from digestion observed in DNMT3B^{WT} cells and to a greater extent in DNMT3B^{W263A} cells. Ethidium bromide stained gel (EtBr) is shown as loading control. Barplot shows signal quantification of satellite II Southern blot using the ratio of the methylated over unmethylated regions indicated. **j)** Genomic location of the two non-repetitive H3K9me3 loci assayed by BS-PCR in this study. H3K9me3 ChIP-seq signals in DNMT3B KO cells are shown above the amplicon locations. ChIP-seq data are the mean of 2 biological replicates. **k-m)** Mean methylation by BS-PCR at H3K9me3 loci alongside the H3K4me3-marked BRCA2 promoter in DNMT3B KO cells expressing DNMT3B mutants. P-values are shown in the figures and from two-

sided Wilcoxon rank sum tests and error bars show the standard error in the man. The number of reads analysed per each sample are shown in *Appendix Table S5* (n is the number of reads in each case). **k)** Expression of catalytically dead DNMT3BW263A does not lead to gain of methylation at H3K9me3-marked loci. **l)** expression of $DNMT3B^{D266A}$ leads to hypermethylation of H3K9me3-marked compared to $DNMT3B^{WT}$. **m)** expression of $DNMT3B^{W263A}$ from the EF1α promoter leads to increased DNA methylation at H3K9me3-marked loci compared to $DNMT3B^{WT}$.

Appendix Figure S3

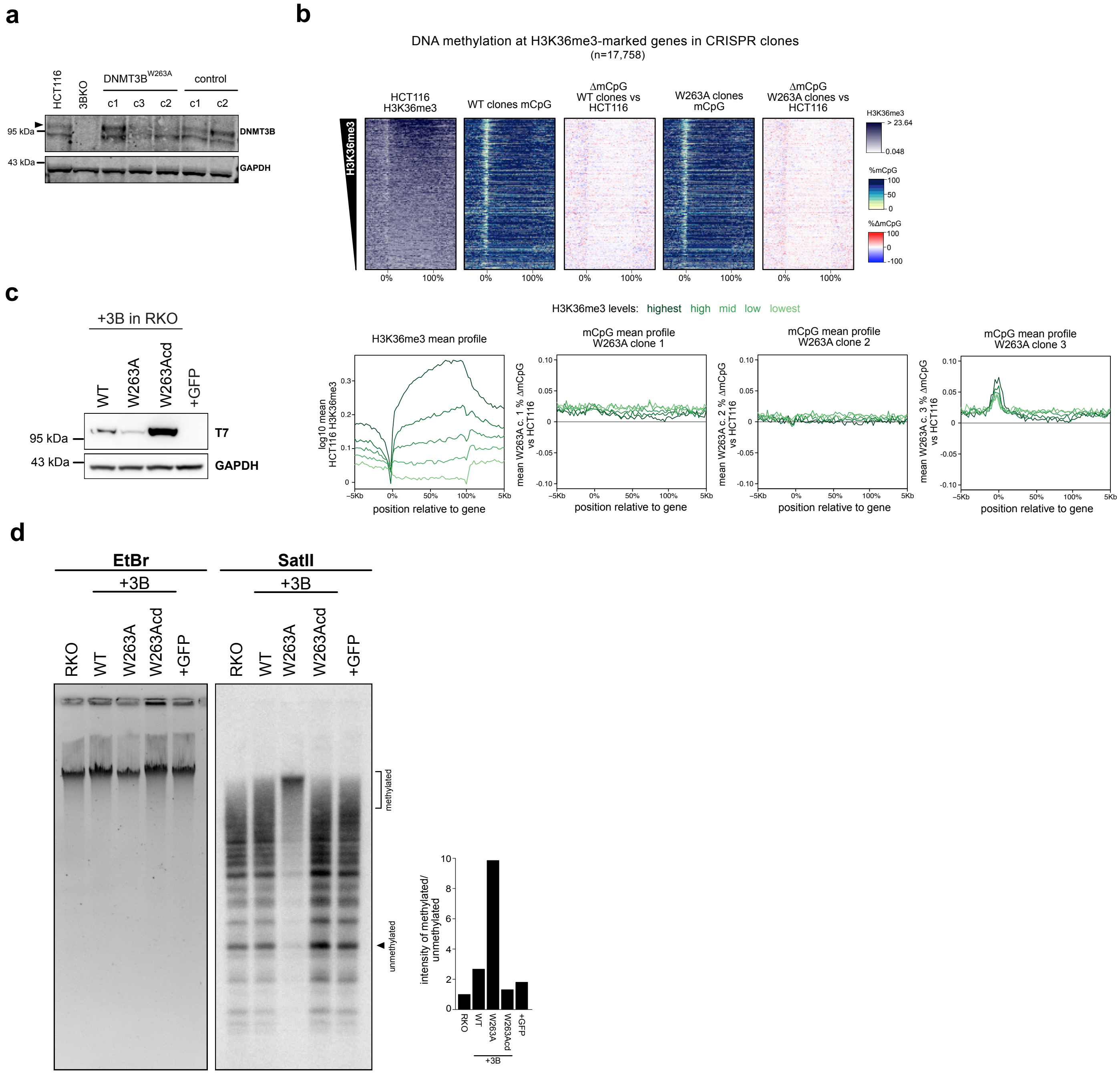

Appendix Figure S3

Knock-in of the W263A mutation causes gains of DNA methylation at heterochromatin

a) Western blot showing DNMT3B expression in CRISPR/Cas9 edited clones. Both DNMT3B2 (black arrow) and the catalytically inactive DNMT3B3 are visible. **b)** H3K36me3 enrichment at gene bodies does not correlate with DNA methylation loss in DNMT3B KO cells. Left, heatmaps showing levels of H3K36me3 and absolute or differential DNA methylation at gene bodies (n=17,758 genes) in HCT116 and DNMT3B CRISPR cells. Genes are ranked by their mean H3K36me3 levels. Values for WT and W263A clones are mean of 2 and 3 clones respectively. Right, profiles of H3K36me3 and differential DNA methylation levels at gene bodies (n=17,758 genes) in the 3 DNMT3B^{W263A} knock-in clones, divided in 5 equally sized groups according to their H3K36me3 enrichment in HCT116 cells. **c)** Western blots showing ectopic expression of T7-tagged DNMT3B proteins or GFP in RKO cells. **d)** Methylation sensitive Southern blot showing reduced digestions of satellite II sequences in DNMT3B^{W263A} cells compared to DNMT3B^{WT} and RKO cells expressing GFP. Ethidium Bromide (EtBr) is shown as loading control. Barplot shows signal quantification of satellite II Southern blot using the ratio of the methylated over unmethylated regions indicated.

Appendix Figure S4

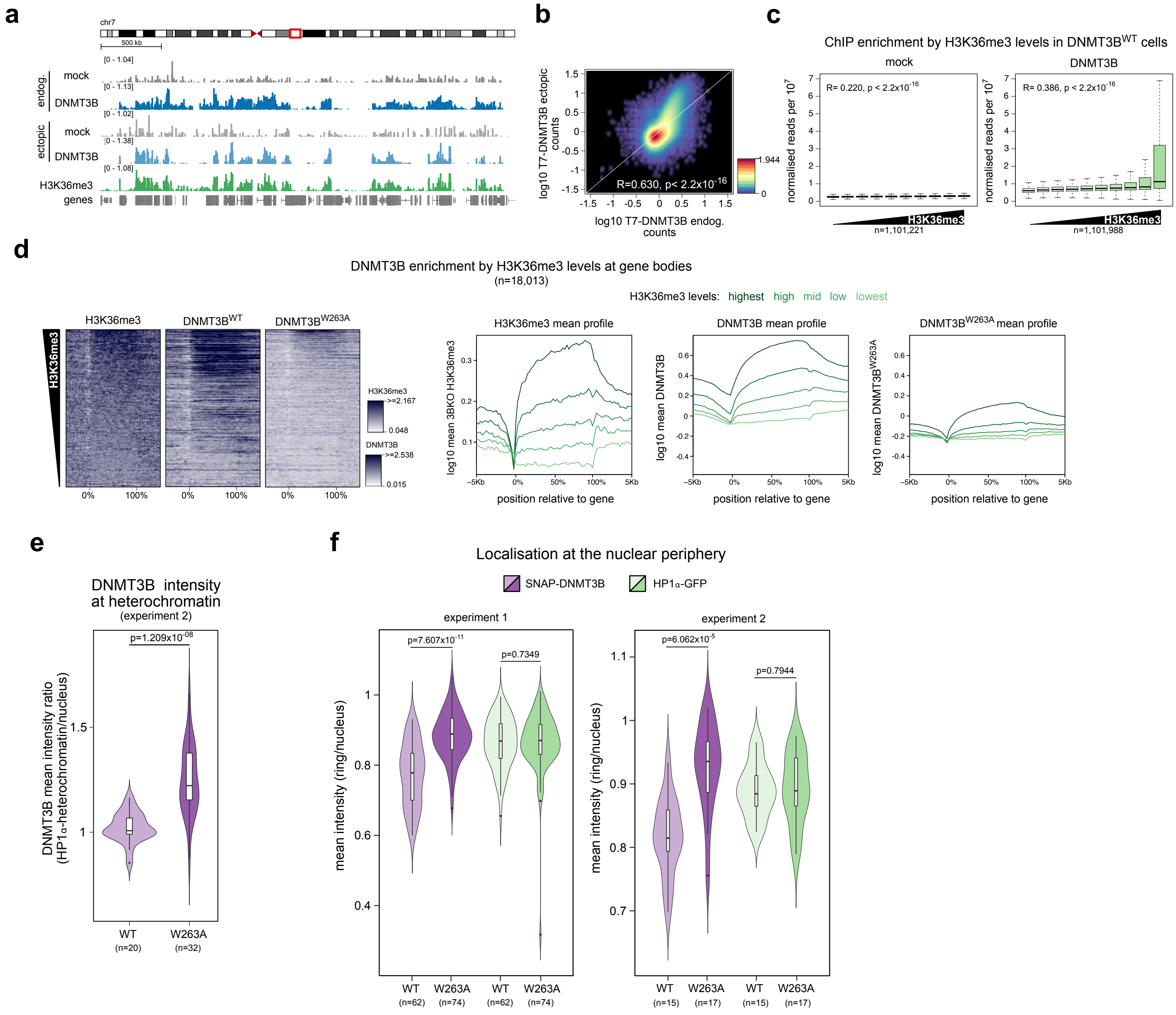

Appendix Figure S4

PWWP mutation leads to increased DNMT3B localisation at heterochromatin

a-c) Ectopically expressed DNMT3B recapitulates the genome binding profile of endogenous DNMT3B. **a)** Binding profiles of endogenous T7-DNMT3B, ectopic T7-DNMT3B and respective mock at a representative genomic region. Genome browser plots showing T7-DNMT3B ChIP signals along with H3K36me3 ChIP-seq in HCT116 cells, normalised reads per 10^6. **b)** Density scatter plot showing genome-wide correlations in 2.5 kb windows (between endogenous T7-DNMT3B and T7-DNMT3B ectopically expressed in HCT116 cells. Correlation was calculated based on log10 transformed normalised read counts. Pearson's correlation (R) and associated p-value is shown. **c)** Boxplot showing mock or T7-DNMT3B^{WT} levels normalised over input at 2.5 kb genomic windows (n=1,101,221 and 1,101,988 windows for mock and DNMT3B respectively) of increased H3K36me3 enrichment in DNMT3B KO cells before being grouped into equally sized deciles by rank. Pearson's correlation, R, and associated p-value is shown. **d)** Decreased enrichment of DNMT3B^{W263A} at H3K36me3 marked gene bodies. Left, heatmaps showing the levels of H3K36me3 in DNMT3B KO cells, T7-DNMT3B^{WT} or T7-DNMT3B^{W263A} at gene bodies (n=18,031 genes). Genes are ranked by their mean H3K36me3 levels. Right, mean profiles of H3K36me3 or T7-DNMT3B proteins levels at gene bodies, divided in 5 equally sized groups according to their H3K36me3 enrichment. **e)** Violin plot showing the distribution of DNMT3B mean intensity ratio between HP1α-marked heterochromatin and the rest of the nucleus from the second biological replicate experiment (first replicate is shown in *Fig. 3e*). **f)** DNMT3B localisation at the nuclear periphery. Violin plot showing the intensity of DNMT3B or HP1α in the 0.5 µm outermost nuclear ring as proportion of the total nuclear intensity. Two independent replicate experiments are shown. For boxplots in **c.** and violin plots in **e.** and **f.**, Lines = median; box = 25th–75th percentile; whiskers = 1.5 × interquartile range from box. P-values in **e.** and **f.** are from two-sided Wilcoxon rank sum tests.

Appendix Figure S5

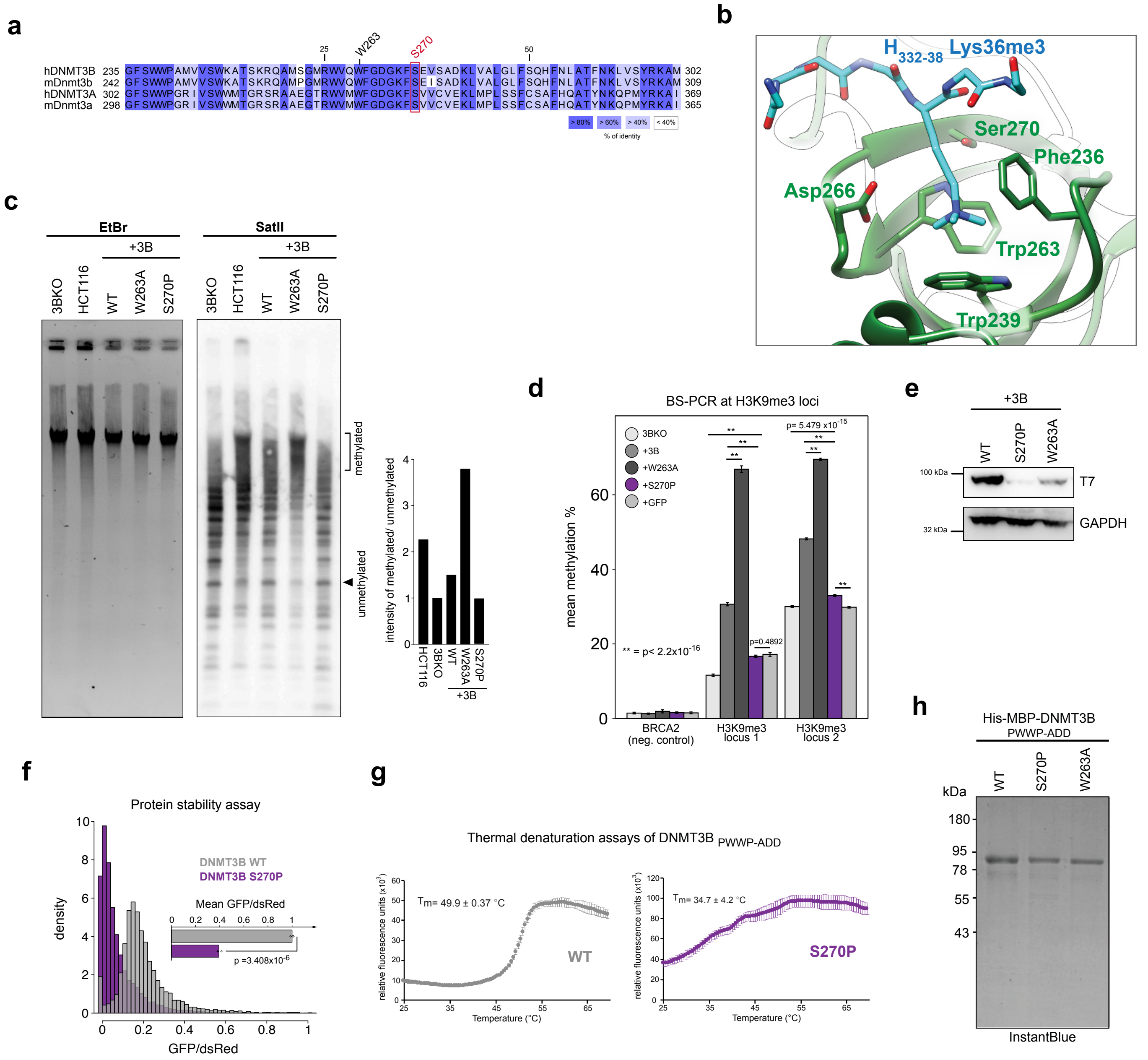

Appendix Figure S5

The ICF syndrome S270P mutation destabilises DNMT3B

a) Multiple sequence alignment of part of the DNMT3B PWWP domain protein sequence with DNMT3A and mouse orthologues showing the location of Serine 270. **b)** Magnified view of DNMT3B histone H3 binding pocket with the ICF1-mutated Ser-270 indicated and H3 Lys 36 shown in blue. **c,d)** $DNMT3B^{S270P}$ cannot remethylate heterochromatin. **c)** Methylation sensitive Southern blot showing digestion of satellite II sequences in DNMT3B KO cells expressing DNMT3B mutants (centre). Ethidium bromide stained gel (EtBr) is shown as a loading control (left). Barplot shows signal quantification of satellite II Southern blot using the ratio of the methylated over unmethylated regions indicated (right). **d)** Mean methylation by BS-PCR at H3K9me3 loci alongside the H3K4me3-marked BRCA2 promoter in DNMT3B mutant cells. P-values are shown in the figure and from two-sided Wilcoxon rank sum tests and error bars show the standard error in the mean. The number of reads analysed per sample is shown in *Appendix Table S5* (n=number of reads in each case). **e-h)** DNMT3B protein carrying S270P mutation is unstable. **e)** Representative western blot showing expression of DNMT3B proteins in DNMT3B KO cells. **f)** Stability of $DNMT3B^{WT}$ and $DNMT3B^{S270P}$ measured by fluorescence reporter. Histogram shows the density distribution of single cell GFP/dsRed ratios of one representative experiment. The barplot shows the mean GFP/dsRed ratios of all the cells, measurements for the mutant normalized to the wild-type, for 3 independent experiments. P value is from Student's T-test. **g)** Thermal denaturation assays on purified $DNMT3B^{WT}$ or $DNMT3B^{S270P}$ protein PWWP and ADD fragments. Graphs showing SYBR-safe fluorescence measured at temperatures from 25°C to 69.5°C. Mean and standard deviation of three technical replicate experiments are plotted with mean Tm values stated. **h)** SDS-PAGE gel of the proteins used in *Appendix Figure S5g* stained with InstantBlue protein stain.

Appendix Figure S6

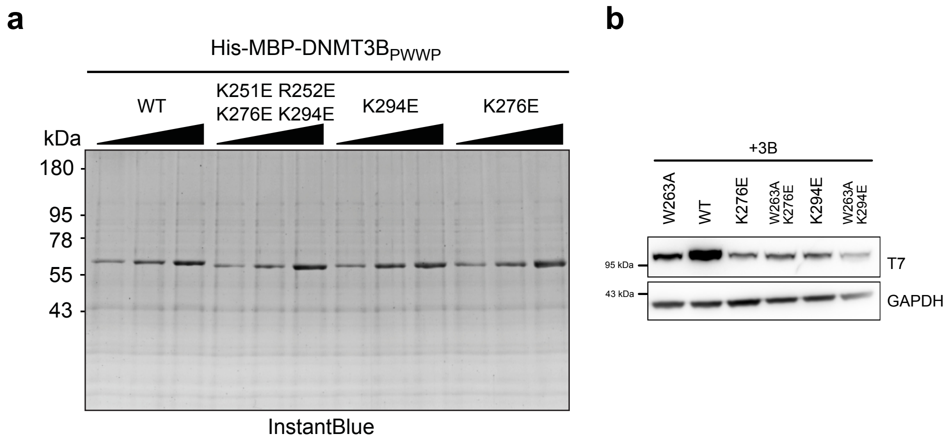

Appendix Figure S6

DNMT3B-PWWP binding to DNA is dispensable for localisation to heterochromatin

a) SDS-PAGE gel stained with InstantBlue protein stain of proteins used in *Fig. 4*. **b)** Western blot of ectopically expressed T7-tagged DNMT3B proteins in DNMT3B KO cells.

Appendix Figure S7

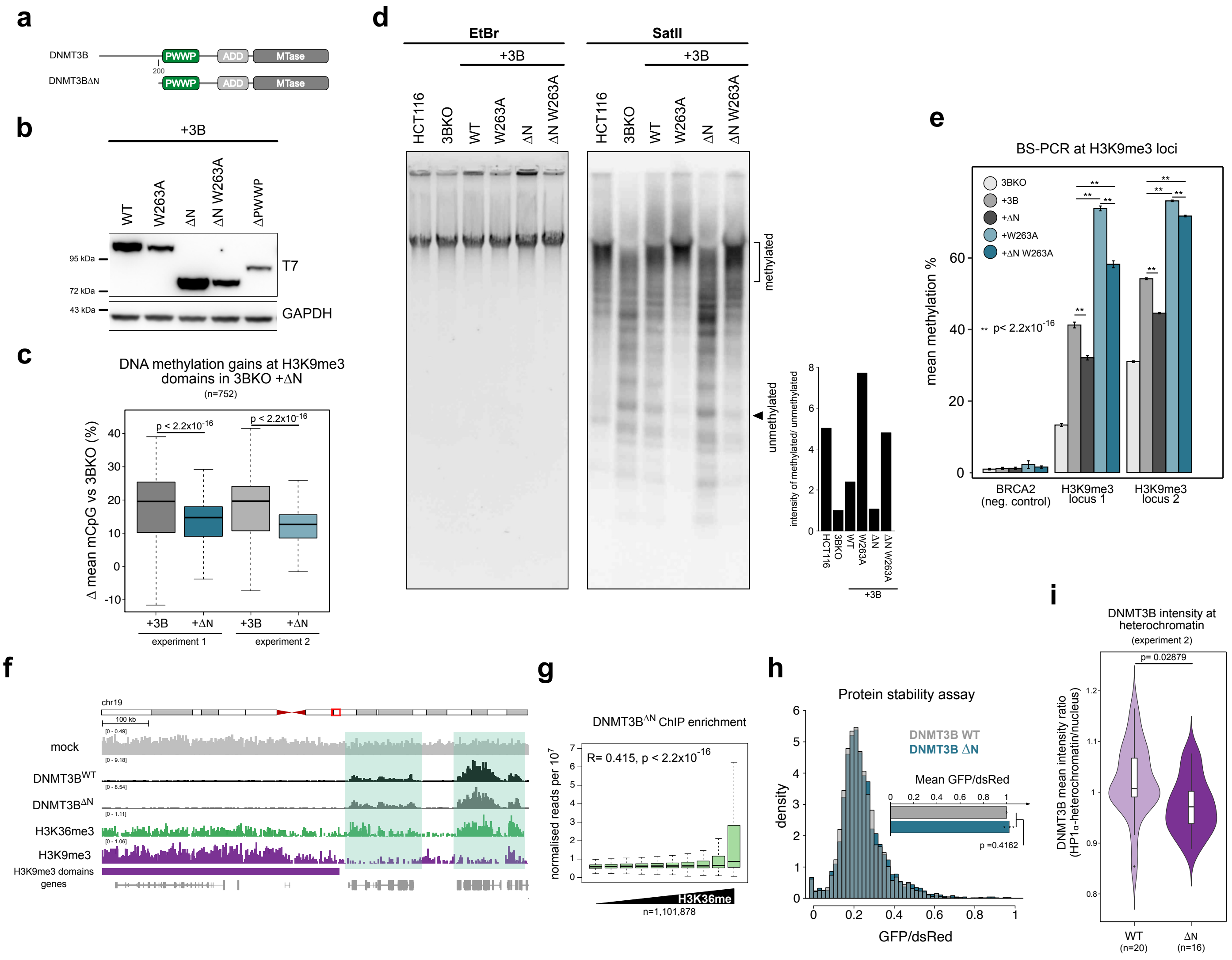

Appendix Figure S7

The N-terminus facilitates methylation of heterochromatin by DNMT3B

a) Schematic of $DNMT3B^{WT}$ and $DNMT3B^{\Delta N}$ proteins. **b)** Western blot of ectopically expressed T7-tagged DNMT3B proteins in DNMT3B KO cells. **c)** Boxplot showing gains of DNA methylation at H3K9me3 domains in $DNMT3B^{WT}$ or $DNMT3B^{\Delta N}$ cells from two biological replicate experiments. Lines = median; box = 25th–75th percentile; whiskers = 1.5 × interquartile range from box. P-values are from two-sided Wilcoxon rank sum tests. **d)** Methylation sensitive Southern blot showing digestion of satellite II sequences in DNMT3B KO cells expressing DNMT3B mutants (centre). Ethidium bromide stained gel (EtBr) is shown as a loading control (left). Barplot shows signal quantification of satellite II Southern blot using the ratio of the methylated over unmethylated regions indicated (right). **e)** Mean methylation by BS-PCR at H3K9me3 loci alongside the H3K4me3-marked BRCA2 promoter in DNMT3B mutant cells. P-values are from two-sided Wilcoxon rank sum tests and error bars show the standard error in the mean. The number of reads analysed per each sample are shown in *Appendix Table S5* (n=number of reads). **f)** Genome browser plots showing $DNMT3B^{WT}$ and $DNMT3B^{\Delta N}$ ChIP signal and H3K36me3 and H3K9me3 ChIP-seq signal from DNMT3B KO cells, normalised reads per 10^6. Green rectangles highlight similar enrichment profiles for $DNMT3B^{WT}$ and $DNMT3B^{\Delta N}$ at H3K36me3-rich loci. ChIP-seq data shown are the mean of two biological replicates. **g)** Boxplot showing levels of T7-$DNMT3B^{\Delta N}$ normalised over input at 2.5 kb genomic windows (n=1,101,878 windows) of increased H3K36me3 enrichment and grouped into deciles of equal sized based on rank. Lines = median; box = 25th–75th percentile; whiskers = 1.5 × interquartile range from box. Pearson's correlation is shown. ChIP-seq data are the mean of two biological replicates. **h)** Stability of $DNMT3B^{WT}$ and $DNMT3B^{\Delta N}$ measured by fluorescence reporter. Histogram shows the density distribution of single cell GFP/dsRed ratios of one representative experiment. The barplot shows the mean GFP/dsRed ratios of all the cells, measurements for the mutant normalized to the wild-type, for three independent experiments. P value is from Student's T-test. **i)** Violin plot showing the distribution of DNMT3B mean intensity ratio between HP1α-marked heterochromatin and the rest of the nucleus from the second biological replicate experiment. For boxplots, lines = median; box = 25th–75th percentile; whiskers = 1.5 × interquartile range from box and p-value are from two-sided Wilcoxon rank sum tests. $DNMT3B^{WT}$ data are repeated from *Appendix Figure S4e* as the data are part of the same experiment.

Appendix Figure S8

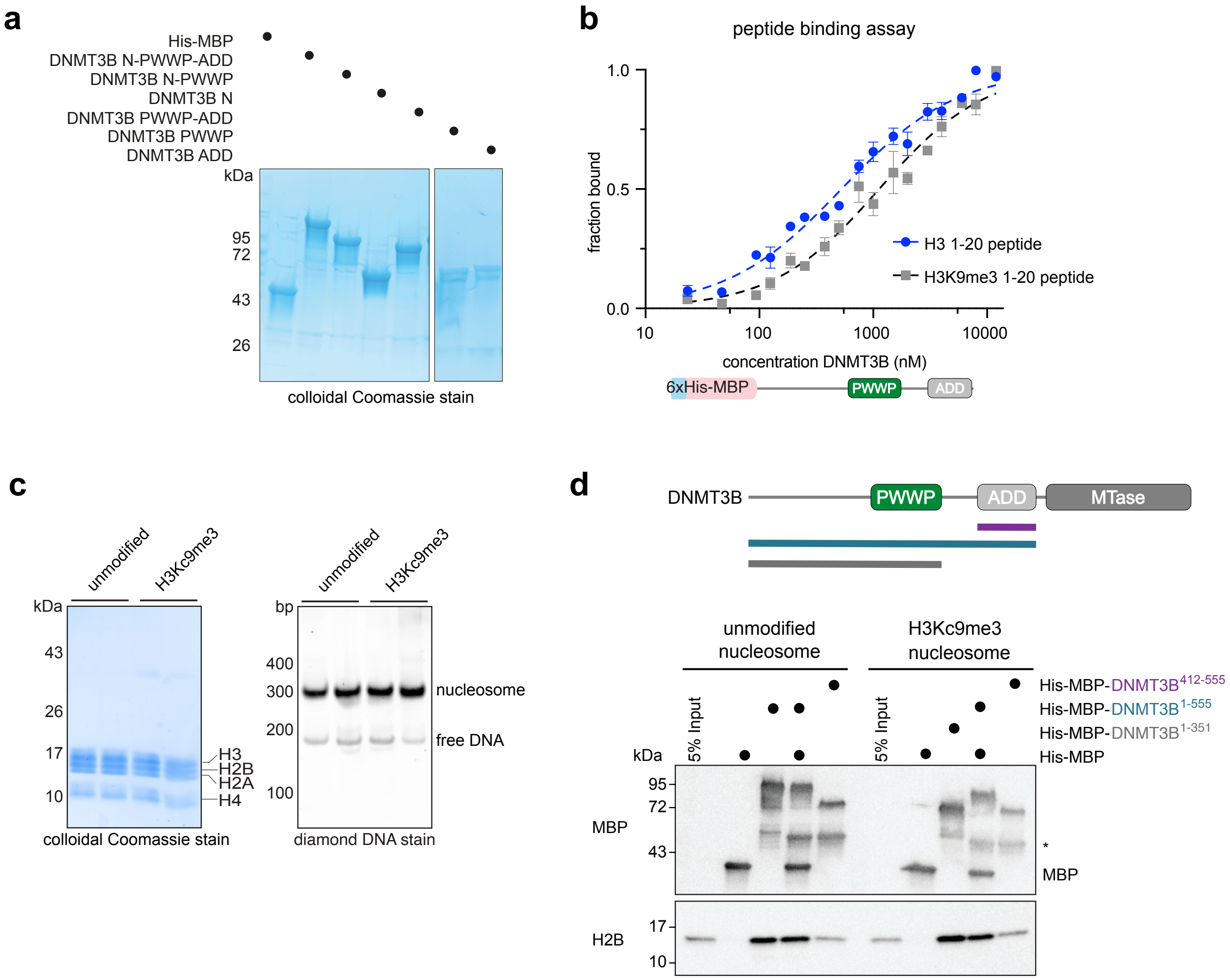

Appendix Figure S8

HP1α interacts with DNMT3B's N-terminal region

a) SDS-PAGE gel showing 6xHis-MBP-DNMT3B fragment proteins used in this figure and *Fig. 6*. Gels were stained with colloidal Coomassie stain. **b)** Graph from fluorescence-stimulation assays showing binding of His-MBP-DNMT3B$^{1\text{-}555}$ to fluorescent peptides. Data was normalised to signal at final saturating concentration and shown as a fraction of peptide bound against protein concentration. Non-linear fit of data shown as dashed lines. Error bars are the standard error in the mane from 3 technical replicates. **c)** Gels showing assembly and quality control of intact nucleosomes. Left, SDS-PAGE gel showing denatured proteins comprising nucleosomes used in this study. Right, native-PAGE gel was stained and imaged with Diamond DNA stain. Shift in mobility suggests DNA is bound and wrapped correctly around octamers. **d)** Immunoblots from pull-down assay using his-MBP-tagged DNMT3B fragments immobilised on amylose affinity beads and incubated with the indicated purified recombinant nucleosomes. For His-MBP-DNMT3B$^{1\text{-}555}$ pull down assays, equal amounts of MBP were also immobilised on the beads (lower band).
